# A Multipoint Correction Method for Environmental Temperature Changes in Airborne Double-Antenna Microwave Radiometers

**DOI:** 10.3390/s140507820

**Published:** 2014-04-29

**Authors:** Jian Sun, Kai Zhao, Tao Jiang

**Affiliations:** 1 College of Electronic Science and Engineering, Jilin University, Changchun 130012, China; 2 Northeast Institute of Geography and Agricultural Ecology, Chinese Academy of Sciences, Changchun 130012, China; E-Mails: zzhaokai@vip.sina.com (K.Z.); jiangtao@neigae.ac.cn (T.J.)

**Keywords:** microwave radiometer, temperature correction method, auto-gain compensation, airborne, double-antenna

## Abstract

This manuscript describes a new type Ka-band airborne double-antenna microwave radiometer (ADAMR) designed for detecting atmospheric supercooled water content (SCWC). The source of the measurement error is investigated by analyzing the model of the system gain factor and the principle of the auto-gain compensative technique utilized in the radiometer. Then, a multipoint temperature correction method based on the two-point calibration method for this radiometer is proposed. The multipoint temperature correction method can eliminate the effect of changes in environmental temperature by establishing the relationship between the measurement error and the physical temperatures of the temperature-sensitive units. In order to demonstrate the feasibility of the correction method, the long-term outdoor temperature experiment is carried out. The multipoint temperature correction equations are obtained by using the least square regression method. The comparison results show that the measuring accuracy of the radiometer can be increased more effectively by using the multipoint temperature correction method.

## 1. Introduction

Microwave radiometers have been widely used in many remote sensing applications in recent decades [1–3]. It is known that while total power radiometers have the simplest structure and measurement scheme, their sensitivity and stability are adversely affected by both noise and gain fluctuation [4]. Although some effective temperature compensation methods have been suggested recently for total power radiometers [5,6], it is still difficult to use them in practical applications. Historically, in order to eliminate the effect of the noise and gain variations, many switched-type radiometers have been developed [7–10], e.g., the Dicke radiometer, the null-balancing Dicke radiometer, the two-reference temperature radiometer, *etc*. However, all of these radiometers use negative feedback control and synchronous detection circuits, which add great complexity and cost to the systems. For this reason, the digital auto-gain compensation microwave radiometer (DGCMR) was proposed [11].

The DGCMR adopts fast analog-to-digital conversion and a software compensation technique, which can compensate the output value of the receiver according to the detected change of the system gain and greatly simplify the system structure. The remote sensing application experiments show that the DGCMR has high sensitivity and stability [12]. However, during the long-term outdoor experiments, we found that the output of the solid-state noise source fluctuates with the environmental temperature changes, which causes measurement errors owing to the digital auto-gain compensation technique. Luan and Zhao analyzed the characteristics of DGCMR and proposed a method of correcting the output value by using the physical temperature of the reference noise source [13]. In the correction procedure, the two-point calibration result can be corrected by the regression relationship between the correction brightness temperature value and the physical temperature of the noise reference source.

In this paper, firstly, a new type Ka-band (frequency is 31.65 GHz) airborne double-antenna microwave radiometer (ADAMR) is introduced. The ADAMR based on the digital auto-gain compensation technique is developed for detecting atmospheric supercooled water content. The detailed results of the sensitivity and stability experiments for this radiometer can be found in [14]. Secondly, we describe the principle of the digital auto-gain compensation technique and analyze the reason for the measurement errors caused by the compensation technique. Then, a multi-point temperature correction method for the two-point calibration equations is proposed. Finally, an outdoor temperature experiment is carried out for the ADAMR. The multi-point temperature correction equations are deduced by using the least square regression method and the experiment results demonstrate the efficiency of the correction method.

## 2. Radiometer Design and Temperature Correction Method

**Figure 1.**
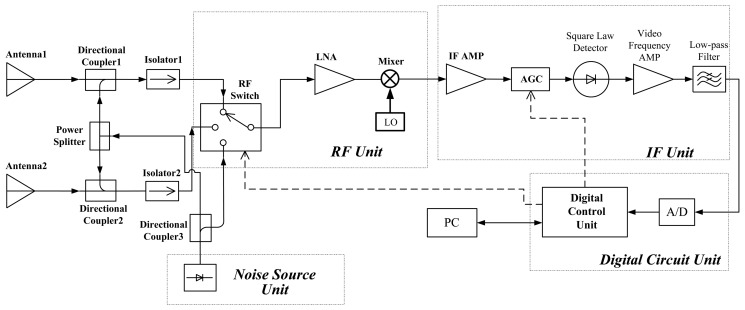
Block diagram of the airborne double-antenna microwave radiometer.

The ADAMR is designed for detecting the radiation brightness temperature from sky. It has been successfully used to retrieve the spatial distribution of SCWC using tomography due to the high sensitivity and the optimized double-antenna structure [15]. The block diagram of the radiometer is shown in Figure 1. The main technical specifications of the receiver and the antenna are listed in the following tables (Tables 1 and 2).

The heterodyne receiver uses a modularization structure, which consists of the radio frequency front end unit (RF unit), intermediate frequency amplifier unit (IF unit), noise source unit and digital circuit unit. In order to detect ultralow (<10 K) effective brightness temperature signals from the sky, the noise coupled technique is used [16]. The thermal noise signal of interest is received by the antennas and coupled into an extra noise signal generated from the solid-state noise source. Then, the noise signal is amplified and down-converted by the RF unit. In the IF unit, the IF amplifier and the auto gain controller (AGC) adjusts the amount of power transferred into the square law detector and the square-law detected voltage signal arrives at a low-pass filter through a differential amplifier, where the high frequency component is excluded. And then, the signal is digitized by a 12 bit A/D converter for auto-gain compensation and further processed in computer.

**Figure 2.**
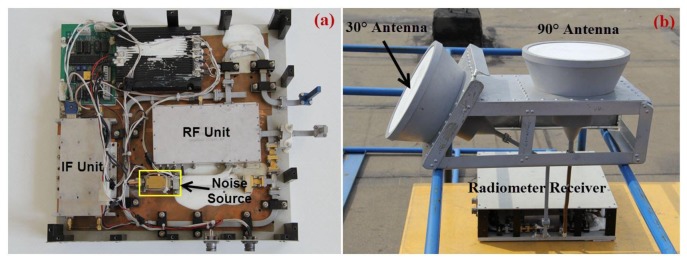
(**a**) The noise source, RF and IF units in the radiometer. (**b**) The radiometer receiver with double antennas in the outdoor experiment.

In order to obtain more observation information, we equip the radiometer receiver with two fixed antennas. The double-antenna elevation angles are 30° and 90°. This has proven to be the best combination of elevation angles according to the results of numerical simulations [17]. Figure 2 shows the main component units of the receiver and the double-antenna structure in the outdoor experiment.

The system gain factor of the radiometer can be expressed as following:

(1) $$G_{S}=g_{LF}g_{DA}C_{d}\text{GkB}$$

where *g_LF_* is the gain of low pass filter, *g_DA_* is the gain of differential amplifier, *C_d_* is the power sensitivity of the square-law detector, *G* is the pre-detection gain, *k* is Boltzmann's constant, *B* is the IF amplifier bandwidth. The RF switch which is controlled by the digital control unit alternately connects the antenna ports and the reference source port to the receiver periodically. In a switch period, the corresponding output voltages of the radiometer when RF switch connects respectively antennas and the reference source can be expressed as:

(2) $$V_{NS}=G_{S}\left(T_{NS}+T_{\text{REC}}\right)$$

(3) $$V_{Ai}=G_{S}\left(T_{Ai}+T_{\text{REC}}\right),(i=1,2)$$

where *T_REC_* is the receiver noise temperature, *T_NS_* is the reference noise source temperature, and *T_Ai_* (*i* = 1,2) is the antenna temperature. When the system gain fluctuates, the corresponding output voltages become:

(4) $$V_{NS}^{\prime }=G_{S}^{\prime }\left(T_{NS}+T_{\text{REC}}\right)$$

(5) $$V_{Ai}^{\prime }=G_{S}^{\prime }\left(T_{Ai}+T_{\text{REC}}\right),(i=1,2)$$

In order to eliminate the effects of the system gain fluctuation, we define a compensation coefficient $\alpha =\frac{V_{NS}}{V_{NS}^{\prime }}$, which can be expressed with Equations (2) and (4):

(6) $$\alpha =\frac{V_{NS}}{V_{NS}^{\prime }}=\frac{G_{S}\left(T_{NS}+T_{\text{REC}}\right)}{G_{S}^{\prime }\left(T_{NS}+T_{\text{REC}}\right)}=\frac{G_{S}}{G_{S}^{\prime }}$$

When *α* ≠ 1, considering the system gain is changed. Through digitizing the voltages and calculating the compensation coefficient by the digital circuit unit and computer, we can real-timely compensate the output voltage of system connecting the antenna by multiplying Equations (5) and (6):

(7) $$V_{Ai}^{\prime \prime }=\alpha V_{Ai}^{\prime }=\frac{G_{S}}{G_{S}^{\prime }}\cdot G_{S}^{\prime }\left(T_{Ai}+T_{\text{REC}}\right)=G_{S}\left(T_{Ai}+T_{\text{REC}}\right),(i=1,2)$$

Comparing Equations (3) and (7), we note that no matter how the system gain changes, the output voltage of the system connecting antenna always keeps constant $V_{Ai}^{\prime \prime }=V_{Ai}$. The observed brightness temperature *T_Ai_* can be estimated by the two-point calibration equation:

(8) $$T_{Ai}=a+bV_{Ai},(i=1,2)$$

where *a* and *b* are the calibration coefficients. However, the auto-gain compensation method mentioned above is based on the assumption that the receiver noise temperature and reference noise temperature are unchanged during the measuring procedure. In fact, a shift in physical temperature of the receiver causing changes in receiver noise temperature and reference noise temperature. Supposing the system gain keeps constant, when the receiver noise temperature and reference noise temperature change, the Equation (2) is transformed to:

(9) $$V_{NS}^{\prime }=G_{S}\left(T_{NS}^{\prime }+T_{\text{REC}}^{\prime }\right)$$

Substituting Equation (8) into Equation (6), we can obtain the gain compensation coefficient:

(10) $$\alpha =\frac{V_{NS}}{V_{NS}^{\prime }}=\frac{T_{NS}+T_{\text{REC}}}{T_{NS}^{\prime }+T_{\text{REC}}^{\prime }}\neq 1$$

Because the compensation coefficient is *α* ≠ 1, the system output voltage will be compensated incorrectly which causes the measuring error in observed brightness temperature. In order to correct this error, deriving from the physical temperature variation of the receiver, we define the measuring error as Δ*T_Ai_*. The corrected antenna temperature $T_{Ai}^{\prime }$ can be expressed as $T_{Ai}^{\prime }=T_{Ai}-\Delta T_{Ai}$. Considering that the fluctuation of the reference noise and receiver noise temperature mainly stem from the temperature drifts of the temperature sensitive components: noise diode in the noise source unit, RF LNA in the RF unit, and square-law detector in the IF unit, we can correct the two-point calibration equation by using the relationship between the measuring error Δ*T_Ai_* and the physical temperatures of the temperature sensitive units above. The internal structure of the radiometer is shown in Figure 2, we can see that all the units of the radiometer are mounted on the same metal baseplate. Taking into account the interaction between the sensitive units, we express the measuring error Δ*T_Ai_* with the multiplication of temperature functions related to each unit as:

(11) $$\Delta T_{Ai}=f_{ns}(T_{NS})\cdot f_{rf}(T_{RF})\cdot f_{if}(T_{IF})$$

where *T_NS_*, *T_RF_* and *T_IF_* are the physical temperatures of the noise source unit, RF unit, and IF unit, respectively. We can expand each of the temperature functions above in a first order power series and retain the second order terms only:

(12) $$\Delta T_{Ai}=a_{1}+a_{2}T_{NS}+a_{3}T_{RF}+a_{4}T_{IF}+a_{5}T_{NS}T_{RF}+a_{6}T_{NS}T_{IF}+a_{7}T_{RF}T_{IF}$$

where the coefficients *a_1_*, *a_2_*, *a_3_*,…*a_7_* can be determined by regressing the result of the temperature experiment using the least square method.

## 3. Temperature Experiment Results

Before the experiment, we calibrated the radiometer system using two-point calibration method which combines the blackbody and the meteorological calibration. In the calibration curve, the high-temperature point is the black body temperature and the low-temperature point is the clear sky brightness temperature collected from a weather station. The calibration equations of the two antenna channels are Equations (13) and (17), respectively. From (UTC +8:00) 11:00 AM, September 22, 2013 to 08:45 AM September 26, 2013, the outdoor temperature experiment for ADAMR was carried out at the Northeast Institute of Geography and Agricultural Ecology, Chinese Academy of Sciences in Changchun City, Jilin Province (East longitude 125°24′, North latitude 43°59′).

**Figure 3.**
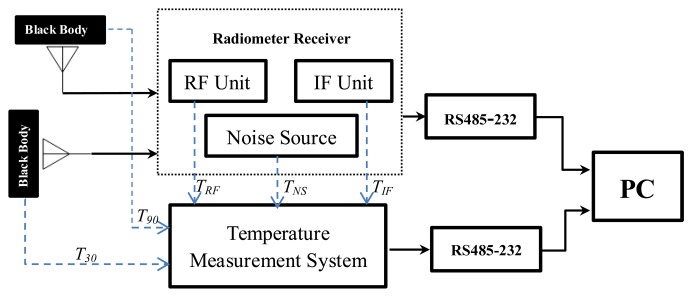
Connection diagram of the outdoor temperature experiment for the radiometer.

**Figure 4.**
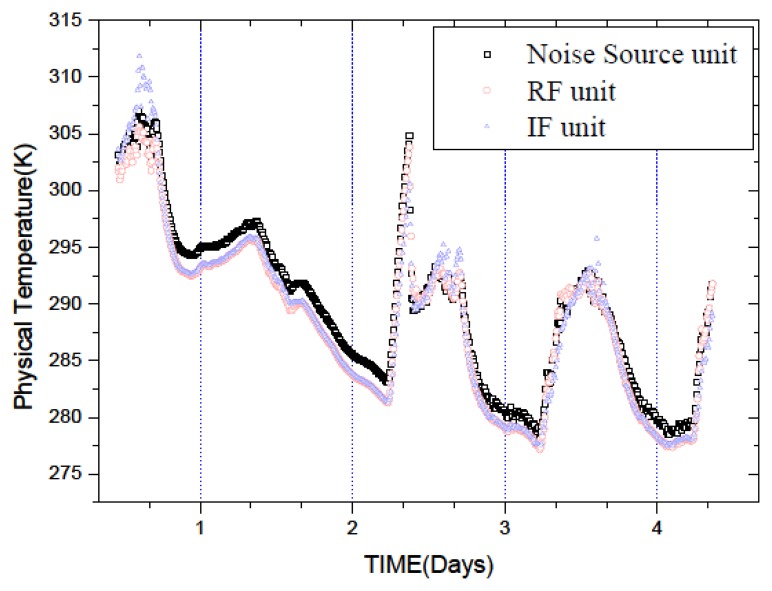
Physical temperature of the noise source, RF and IF units during the temperature experiment.

The connection block diagram of this temperature experiment is shown in Figure 3. During the experiment, the two antenna apertures are covered by two pieces of microwave black body material, respectively, as the observed target. The temperature measurement system collects the temperatures of the observed target, the noise source, the RF, and the IF units by the temperature probes (DS18B20). Then the recorded temperature and the radiometer output voltage data are transferred real-timely to PC via a RS485-232 serial communication interface for further processing. Figure 4 shows the temperatures of the noise source, the RF, and the IF units recorded by the temperature measurement system. Due to the large size of the data, we take the accumulative average value per minute as the measurement value of this minute.

### 3.1. Results of 30° Antenna Channel

**Figure 5.**
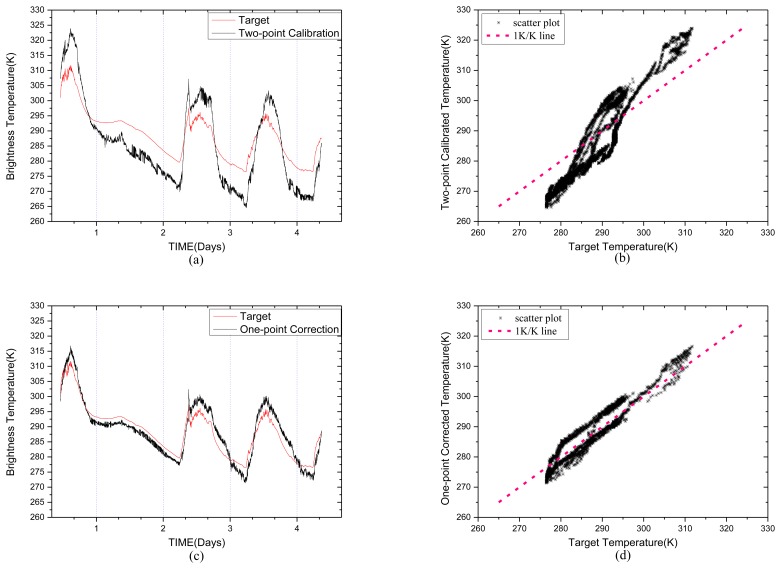

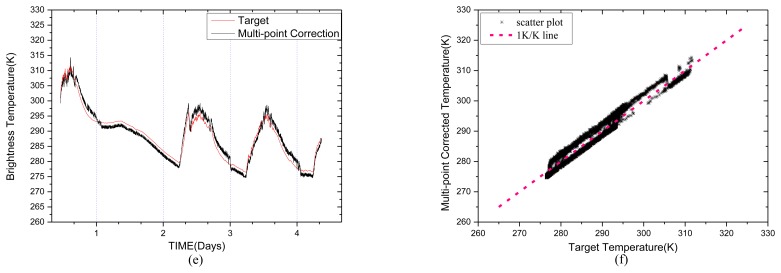
The estimated temperatures for the 30° antenna using different methods compared with the blackbody target temperature. (**a**) Two-point calibration method. (**b**) Scatter diagram of the two-point calibration. (**c**) One-point correction method and (**d**) Scatter diagram. (**e**) Multi-point correction method and (**f**) Scatter diagram.

The temperature of the target ranges from 276 K to 311 K. The uncorrected two-point calibration equation is:

(13) $$T_{B30}=-369.4747+0.2932V_{30}$$

where *V_30_* is the output voltage from the 30° antenna channel, *T_B30_* is the corresponding brightness temperature of the observed target. The black body target physical temperature and the brightness temperature estimated by the two-point calibration method are shown in Figure 5a. As can be seen, the two-point calibrated brightness temperatures have poor consistency with the target temperatures. The measurement error is considered as the difference between the target temperature and the calibrated temperature, in other words, Δ*T_B30_* = *T_target_* − *T_B30_*.

We obtain the relationship between the temperature of reference source *T_NS_* and the measurement error Δ*T_B30_* by using the 2nd-order polynomial fit method:

(14) $$\Delta T_{B30}=-993.8652+5.6165T_{NS}-0.0076T_{NS}^{2}$$

The one-point temperature correction equation can be expressed as:

(15) $$T_{B30}^{\prime }=624.3905+0.2932V_{30}-5.6165T_{NS}+0.0076T_{NS}^{2}$$

Figure 5c shows the one-point corrected temperature curve. We can see that the one-point correction method can compensate the effect caused by the variation of the environment temperature to a certain extent compared with the two-point calibration method. The RMSE of two-point calibration temperature is 7.3798 K, while the RMSE of the one-point corrected temperature is 3.0306 K. Figure 5b,d show the scatter points of the estimated temperature using the two methods above respectively. The correlation coefficients are 0.945 in Figure 5b and 0.952 in Figure 5d.

We use the temperatures of the units shown in Figure 4 and the measurement error to extract coefficients of the multi-point temperature correction equation by least squares method. The corrected calibration equation is:

(16) $$T_{B30}^{\prime \prime }=-136.7254+0.2932V_{30}-26.2946T_{NS}+74.9739T_{RF}-49.0660T_{IF}-0.1585T_{NS}T_{RF}+0.2688T_{NS}T_{IF}-0.1119T_{RF}T_{IF}$$

The results of the corrected calibration are shown in Figure 5e,f. The RMSE is 1.8426 K, and the correlation coefficient is 0.9764.

### 3.2. Results of 90° Antenna Channel

**Figure 6.**
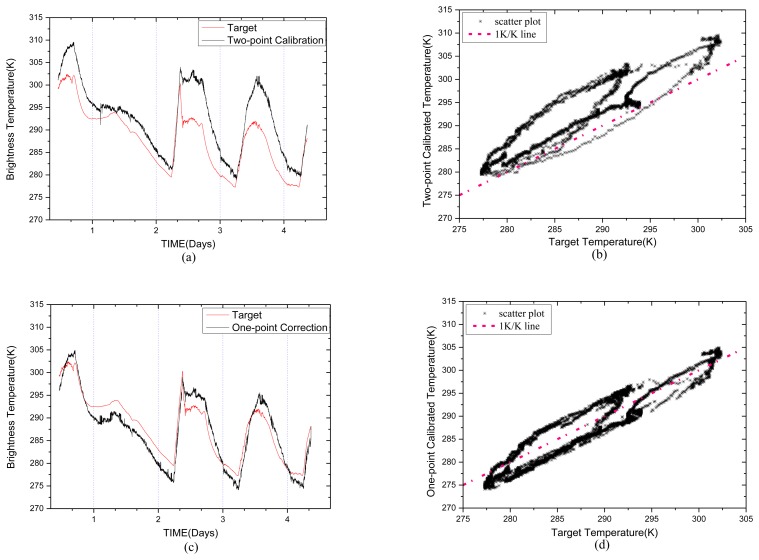

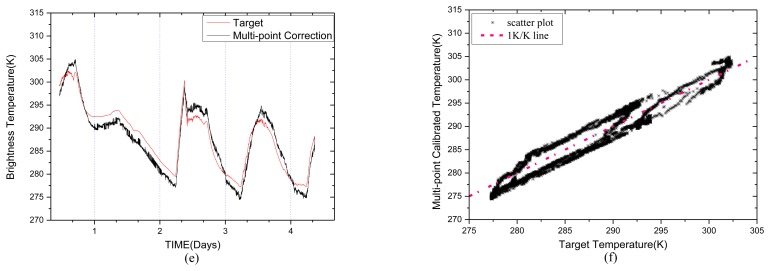
The estimated temperatures for the 90° antenna using different methods compared with the blackbody target temperature. (**a**) Two-point calibration method. (**b**) Scatter diagram of the two-point calibration. (**c**) One-point correction method and (**d**) Scatter diagram. (**e**) Multi-point correction method and (**f**) Scatter diagram.

The black body target physical temperature and the brightness temperature estimated by the two-point calibration method are shown in Figure 6a. The temperature of the target ranges from 277 K to 302 K. The uncorrected two-point calibration equation is:

(17) $$T_{B90}=-39.0528+0.1511V_{90}$$

The relationship between the temperature of reference source *T_NS_* and the measurement error Δ*T_B_*_90_ is:

(18) $$\Delta T_{B90}=-451.0885+3.1417T_{NS}-0.0054T_{NS}^{2}$$

The one-point temperature correction equation can be expressed as:

(19) $$T_{B90}^{\prime }=412.0357+0.1511V_{90}-3.1417T_{NS}+0.0054T_{NS}^{2}$$

Figure 6c shows the curve of one-point corrected temperature. The RMSE of two-point calibration temperature is 5.6248 K, while the RMSE of the one-point corrected temperature is 2.6740 K. Figure 6b,d show the scatter points of the estimated temperature using the two methods above respectively. The correlation coefficients are 0.918 in Figure 6b and 0.943 in Figure 6d.

We can extract coefficients of the multi-point temperature correction equation by least squares method using the temperatures of the units and the measurement error. The corrected calibration equation is:

(20) $$T_{B90}^{\prime \prime }=466.6771+0.1511V_{90}+13.1716T_{NS}+21.4678T_{RF}-38.3093T_{IF}-0.1168T_{NS}T_{RF}+0.0802T_{NS}T_{IF}+0.0432T_{RF}T_{IF}$$

The results of the corrected calibration are shown in Figure 6e,f. The RMSE is 2.0433 K, and the correlation coefficient is 0.965.

## 4. Conclusions

This paper introduces a new type Ka-band airborne double-antenna microwave radiometer for detecting atmospheric supercooled water content. Although the auto-gain compensative technique can eliminate the effect of the system gain fluctuation and greatly simplify the structure of the radiometer receiver, it also imports measurement errors when the radiometer operates in varying temperature environments. For this reason, we propose a new multi-point temperature correction method and carried out a long-term outdoor temperature experiment with the radiometer. The results demonstrate that the temperatures of the two antenna channels corrected by using this method have higher correlation with the target temperatures than the uncorrected two-point calibration and the one-point temperature correction methods. Since the brightness temperature is the key parameter for retrieving SCWC, it is important to obtain highly accurate flight observation brightness temperature data. This new multipoint correction method will be able to eliminate the effect of the dramatic changes caused by the aircraft cabin temperature on the airborne radiometer and improve SCWC retrieval accuracy effectively in further flight observation experiments.

## Figures and Tables

**Table 1.** The main technical specifications of the radiometer receiver.

**Frequency (GHz)**	**Bandwidth on IF (MHz)**	**Noise Figure (dB)**	**Sensitivity (K)**	**Linearity**	**Integration Time (ms)**	**Range of Measurement Temperature (K)**	**Stability (K)**
31.65	400	≤5	≤0.2	r ≥ 0.9995	300	10–350	≤1

**Table 2.** The parameters of the radiometer antenna.

**Antenna Type**	**Antenna Aperture (mm)**	**Antenna Gain (dB)**	**3 dB Beam Width (deg)**	**Antenna Sidelobe Level (dB)**	**VSWR**
Dielectric Lens	250	≥32	4.2	≤ −32	≤1.3
